# Impact of Thyme Essential Oil on the Aroma Profile and Shelf Life of Vacuum-Packed Minced Turkey Meat

**DOI:** 10.3390/molecules29153524

**Published:** 2024-07-26

**Authors:** Paweł Satora, Magdalena Michalczyk, Joanna Banaś

**Affiliations:** 1 Department of Fermentation Technology and Microbiology, Faculty of Food Technology, University of Agriculture in Krakow, Balicka 122, 30-149 Krakow, Poland; 2 Department of Biotechnology and General Technology of Food, Faculty of Food Technology, University of Agriculture in Krakow, Balicka 122, 30-149 Krakow, Poland; joanna.banas@urk.edu.pl

**Keywords:** aroma constituents, principal component analysis, poultry meat, lipid oxidation

## Abstract

There is considerable interest in the use of essential oils for food preservation, but their effect on the aroma profile of a product is poorly understood. This study investigated the effect of thyme essential oil (EO) addition at increasing concentrations (0.005, 0.01, 0.02, and 0.03% *v*/*w*) on the volatile compound composition of vacuum-packed minced turkey meat after storage for 8 days at 1–2 °C. The aroma profile of the meat was determined using the HS-SPME/GCMS (headspace solid-phase microextraction/gas chromatography–mass spectrometry) method. The results were also analysed by PCA (principal component analysis). The addition of thyme EO had a modifying effect on the aroma profile of meat-derived components, e.g., the formation of benzeneacetaldehyde, benzyl alcohol, 4,7-dimethylbenzofuran, hexathiane, hexanal, and 1-hexanol was reduced and the appearance of 9-hexadecenoic acid was observed in the stored samples. The increase in EO concentration affected the levels of its individual components in the meat headspace in different ways. In terms of fat rancidity indices, even a 0.005% addition of this essential oil significantly reduced the peroxide value. Quantitative descriptive analysis (QDA) showed that the addition of thyme EO reduced or masked the intensity of unpleasant odours associated with meat spoilage. In the aroma analysis, the turkey with 0.02% *v*/*w* EO scored highest, and pleasant citrus notes were found.

## 1. Introduction

Minced meat is one of the most perishable meat products. This meat obtained from turkey is relatively rarely used as a raw material in studies on additive–meat interactions. This is undoubtedly due to the much greater popularity of minced beef and pork and the wide range of products made from them. However, there are now proposals to reduce the consumption of ruminant meat in favour of poultry meat [1]. Font-i-Furnols [2] reports that between 2010 and 2020, a decrease in red meat consumption and an increase in poultry meat consumption was observed among consumers in many countries. As a result, there is likely to be a growing interest in turkey products, including those derived from minced meat. Such products can be enriched, modified, and preserved with various additives of plant origin, including extracts and essential oils.

Odour is one of the basic criteria for the evaluation of raw meat. The components of odour are mainly derived from the transformation of fats, proteins, and carbohydrates due to oxidation and the activity of both endogenous enzymes and microorganisms [3,4,5]. Furthermore, the additives utilised also influence the odour of meat products. Thyme, which is considered a spice or medicinal plant, is one of the most popular herbs used in the kitchen. In traditional medicine, thyme is known to possess anti-inflammatory, antispasmodic, analgesic, expectorant, antibacterial, and antifungal properties [6]. Thymol, the primary constituent of thyme oil, exhibits anti-inflammatory, antioxidant, anti-hyperlipidemic, and antimicrobial properties. Furthermore, its protective effects in metabolic and neurodegenerative disorders and gastrointestinal diseases have been observed in animal and cellular studies. In cell line studies, the potential of thymol as an anticancer agent was determined, among other factors, by its ability to induce apoptosis and inhibit proliferation and angiogenesis [7]. The potential use of thyme essential oil (EO) in animal nutrition is also being investigated. In the case of Japanese quails, the impact of the essential oil of thyme on feed intake, serum creatinine, low-density lipoprotein, serum magnesium levels and liver catalase, superoxide dismutase, and glutathione peroxidase activities was demonstrated [8]. Furthermore, the authors observed a reduction in liver and serum lipid peroxidation in the birds fed the EO of thyme.

The essential oil from this plant, which has been traditionally used for flavouring and preserving food, has been examined for its potential to preserve various food products due to its antimicrobial properties. These include the possibility of using it at a concentration of 1% (*v*/*w*) to preserve vacuum-packed chicken breast muscle exposed for 1 min, as well as its potential usefulness as a natural food preservative [9]. As reported by Thanissery and Smith [10], the utilisation of essential oils derived from thyme and orange in marinade has been demonstrated to reduce the levels of *Salmonella Enteritidis* and *Campylobacter coli* in broiler breast fillets and whole wings. Saricaoglu and Turhan [11] conducted a study in which they tested mechanically deboned chicken meat protein coatings containing essential oils of thyme or clove to improve the quality of stored slices of traditional Turkish-style fermented sausages that had undergone heat treatment. In both cases, the application of the coatings was observed to have a beneficial effect; however, the improvement was more pronounced in the case of the coating containing the essential oil of clove. In a separate study, Boskovic et al. [12] investigated the impact of essential oils of thyme and oregano at concentrations ranging from 0.3% to 0.9% on the oxidative stability of minced pork. The authors found that both oils had a beneficial effect on reducing fat oxidation in samples stored for two weeks.

The potential beneficial effect of essential oils added to meat depends on their type and concentration, which is primarily limited by sensory acceptability. The addition of essential oils to meat has a significant impact on the aroma of the resulting product. Consequently, both the concentration and type of added EOs must be chosen appropriately for the type of meat product. Studies on the influence of EOs on meat stability have mainly evaluated the antimicrobial and antioxidant efficacy of such additives [13,14,15,16]. However, little attention has been paid to detailed studies of the changes in individual components of meat odour that occur with EOs. This issue is of interest not only because of the aroma formation of stored products, but also because of the stability assessment of individual bioactive components of essential oils during food storage.

The aim of this study was to evaluate the influence of varying concentrations of thyme EO on the volatile compound profile of minced turkey meat stored at 1–2 °C. The hypothesis was that the components of essential oils may affect the formation of volatile components associated with the odour of perishable meat. A range of additive concentrations was employed, which, according to the available literature, were among the lowest examined in previous studies [17,18]. A reduction in the quantity of added flavouring substances may have a positive impact on consumer acceptance. The essential oil of thyme was selected for its documented antioxidant and antimicrobial properties, as well as the herb’s high popularity.

## 2. Results and Discussion

### 2.1. Essential Oil Volatile Compounds Evaluation

Table 1 presents the relative quantity of aroma components in the essential oil of thyme. A total of 26 volatile components were identified, with thymol being the most abundant, followed by borneol, caryophyllene, γ-terpinene, α-terpineol, p-cymene, linalool, carvacrol, and α-terpinene. Hudaib and Aburjai [19] reported that dried cultivated or wild *Thymus vulgaris* L. contained between 1.1 and 5.6% essential oil, with higher values for wild plants. The authors identified the principal constituents of the examined products as phenolic monoterpenoids, including thymol and carvacrol, in addition to smaller concentrations of p-cymene, γ-terpinene, 1,8-cineole, α-thujone, camphor, and β-caryophyllene. The isolation yield and chemical composition of EOs are influenced by a number of factors, including the environment, the region of growth, and the cultivation practices employed [19].

In order to minimise the impact of the matrix (turkey meat) on the chromatographic analysis, samples were analysed immediately following the introduction of EO of thyme (at different concentrations) and after an 8 day storage period. Table 2 illustrates the differences in the content of individual essential-oil-derived components, with colours reflecting an increase in the amount of a given component during storage (various shades of green) or a decrease in its level (shades of red). The concentration of a given compound in the meat after storage is expressed as a numerical value.

Meat storage has little or no effect on the concentration of a small group of EO components such as thymol, the dominant compound in thyme EO, but also borneol, terpinene-4-ol, or p-cymenene (Table 2). The largest group of compounds were those whose content decreased after storage, and this is particularly evident in the case of highly volatile compounds such as α-thujene, α-pinene, camphene, and β-pinene. A similar trend was observed for some of the less volatile components (LRI 1376-1514) (Table 2). As noted by da Silva et al. [20], EO components may undergo oxidation, polymerisation, and volatilisation when incorporated into the meat matrix. In addition, essential oil components can be used by microorganisms as a carbon and energy source [21]. Other reactions that may influence changes in the concentrations of EO components are hydrogenation or dehydrogenation and rearrangement [22]. The above-mentioned transformations could have influenced the formation of some components during storage or could have led to an increase in their levels observed in this study. Such a tendency can be observed for cis-linalool oxide, caryophyllene alcohol, and caryophyllene oxide, whose precursors are linalool and caryophyllene [23,24]. An example of such a compound is also carvone (Table 2). Salim et al. [22] observed a similar phenomenon of carvone formation during storage of spearmint oil. This component may be formed by microbial transformation of D-limonene [21].

Another phenomenon was also observed after the addition of thyme EO to turkey meat. The content of constituents such as α-pinene, camphene, and γ-terpinene increased with an increase in the concentration of EO added up to the level of 0.02%; its further increase did not lead to an increase in the concentration of the constituent. This phenomenon could be related to interactions with lipophilic components of the meat matrix, high volatility, and limited solubility of these components in the aqueous phase [25,26]. Above a certain limit, the remaining introduced compounds remained in an unbound/undissolved form and were released during storage or when the packaging was opened. For components such as linalool, terpinene-4-ol, α-terpineol, and thymol, an increase in the concentration of EO in the meat also led to an increase in their concentration in the samples. The above observations suggest, among other things, that it is possible to obtain different flavour profiles by using different oil concentrations and also after storage.

### 2.2. Changes in the Content of Volatile Components from Turkey Meat during Storage

The sum of volatiles derived from meat ranged from 356.4 (addition of 0.01% EO) to 530.4 μg·kg^−1^ (addition of 0.03% EO). A similar value of total volatile compounds obtained by simultaneous distillation and extraction, 536.1 μg·kg^−1^ of raw chicken breast meat, is reported by Ayseli et al. [27]. The data obtained in this study do not indicate the occurrence of a high concentration of volatiles in stored meat. This proves that the formation of the intense odour in perishable meat was not associated with an increase in the content of volatile compounds, but rather with the formation of compounds with a lower odour threshold or with a negative effect on odour. Some variation in the sum of compounds in individual samples may be due to different proportions of substances with different molecular weights in them. A similar phenomenon was described by Song et al. [28], who studied aroma compounds at four stages of tallow oxidation. The authors observed a slight decrease in the concentration of volatile compounds at the early oxidation stage, which they attributed to the formation of hydroperoxide at this stage, but also to the influence of polymerisation and other reactions, including oxidation. As the oxidation process progressed, Song et al. [28] observed first a significant increase and then some decrease in the concentration of volatile compounds.

The results of the analysis of volatile components derived from turkey meat are presented in Table 3. For some compounds, the effect of the added essential oil on their formation after the storage period was observed to be limiting. This was the case, for example, for benzeneacetaldehyde, benzyl alcohol, 4,7-dimethylbenzofuran, ethyl 2-methyloctanoate, and hexathiane. In the case of some low-molecular compounds such as, for instance, 3-propoxy-1-propene, 1-pentanol, hexanal, nonanal, heptenal, octenal, 1-hexanol, and 2-pentylfuran, their content in the control sample decreased after storage. The addition of the EO resulted in a further reduction in their concentration. With regard to the concentration of dodecanoic acid, ethyl dodecanoate, ethyl tetradecanoate, and ethyl hexadecanoate, the addition of the essential oil resulted in an increase in their concentration following storage, in comparison to the control. 9-Hexadecenoic acid was observed only in samples that had been stored and contained essential oil. Astudillo et al. [29] have demonstrated that this fatty acid exerts anti-inflammatory effects even at low concentrations. Nevertheless, its role in the human body remains incompletely elucidated, whereas its action as a lipid hormone has been observed in animal models.

As previously stated, benzeneacetaldehyde was among the substances whose concentration in the examined meat stored without EO exhibited a marked increase, while the addition of EO resulted in a reduction in this increase. Both benzaldehyde and benzeneacetaldehyde can be formed as a consequence of microbial spoilage [4]. In our previous study, we also found no benzeneacetaldehyde in fresh meat and that the formation of this substance was reduced in stored products with caraway and rosemary essential oils [30]. The presence of ethyl acetate, dimethyl disulfide, benzeneacetaldehyde, n-decanoic acid, and 2-methyldecanoic acid was observed exclusively in the stored products. According to Feng et al. [31], dimethyl disulfide was absent in fresh turkey meat, but it appeared after irradiation of meat. This component has a very low threshold, much lower than other compounds, and is characterised by a strong and stringent odour. As Song et al. [26] report, benzaldehyde is an important component of unoxidised tallow. On the other hand, (E,E)-2,4-heptadienal, (E,E)-2,4-decadienal, E-2-nonenal, octanal, hexanoic acid, hexanal, and (E)-2-heptenal are the main odour components formed during tallow oxidation. At the same time, the authors [28] observed a decrease in the concentration of hexanal, heptanal, 2-pentylfuran, octanal, and nonanal during the initial stages of tallow oxidation, a finding that is consistent with our own observations. With regard to turkey meat, the presence of EO resulted in an additional strong reduction in the concentration of the above-mentioned substances in the vast majority of cases.

It is evident that as the concentration of EO increases, the undesirable changes in meat odour are masked to a greater extent. Nevertheless, notable alterations (decrease or/and increase) in the content of certain compounds following the addition of EO, in comparison to the control sample (Table 3), may have a considerable impact on the odour of stored meat. For example, the EO concentrations of 0.01% and above were found to result in a reduction in the concentration of hexanal and nonanal below their OAVs (odour activity values). A similar phenomenon was observed in the case of 1-octen-3-one and benzeneacetaldehyde at EO additions of 0.02% and above. In contrast, the levels of acetaldehyde and decanal increased above their OAVs [32] in the presence of thyme EO. It is often challenging to ascertain precisely which compounds have exceeded their OAV, in part due to the considerable discrepancies in the ranges of reported odour thresholds across the literature. At the same time, it should be noted that not every detected substance has an odour threshold reported in the literature. Based on the OAV [32], it can be assumed that the odour-active components of raw, fresh turkey minced meat are hexanal, heptanal, dimethyl trisulfide, 1-octen-3-one, 2-pentylfuran, octanal, 2-octenal, nonanal, and decanal (mainly aldehydes). Conversely, the aroma of meat stored without EO was predominantly influenced by dimethyl disulfide, hexanal, octanal, dimethyl trisulfide, benzeneacetaldehyde, 1-octen-3-one, octen-3-ol, nonanal, decanal, and dodecanal. However, the compounds present in both fresh and stored turkey meat often occurred in concentrations that varied several times.

**Figure 1:**
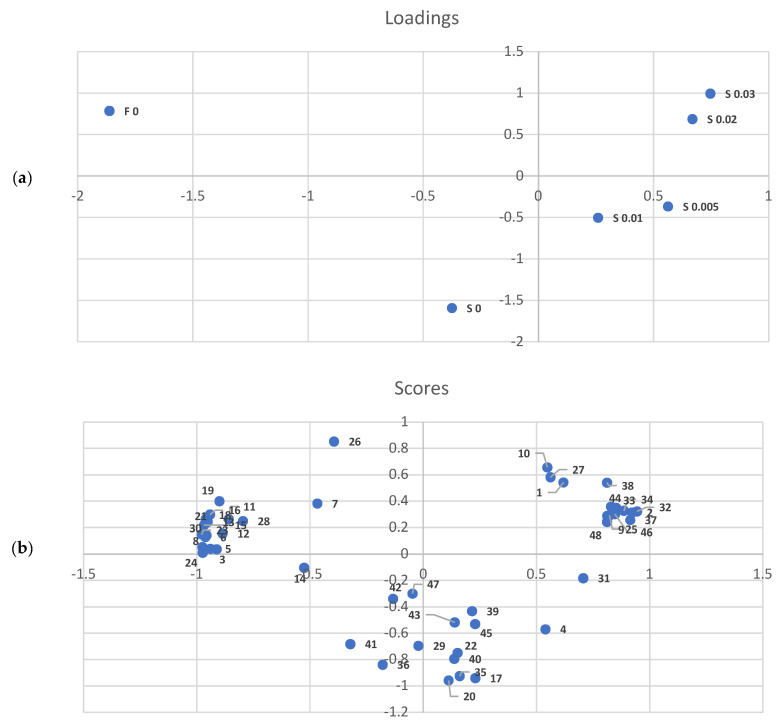
Principal component analysis (PCA) scores and loadings plots of the volatile compounds of the turkey meat after the addition of different concentrations of thyme essential oil. (**a**) F—fresh meat; S—stored meat; 0.005, 0.01, 0.02, 0.03—percentage concentration of thyme essential oil in sample. (**b**) 1—acetaldehyde; 2—ethyl acetate; 3—3-propoxy-1-propene; 4—dimethyl disulfide; 5—1-pentanol; 6—hexanal; 7—4-ethylbenzamide; 8—1-hexanol; 9—butyrolactone; 10—dimethyl sulfone; 11—heptanal; 12—benzaldehyde; 13—2-heptenal; 14—dimethyl trisulfide; 15—1-octen-3-one; 16—octen-3-ol; 17—hexanoic acid; 18—2-pentylfuran; 19—octanal; 20—benzeneacetaldehyde; 21—2-octenal; 22—benzyl alcohol; 23—2-octen-1-ol; 24—nonanal; 25—octanoic acid; 26—ethyl octanoate; 27—dimethyl tetrasulfide; 28—decanal; 29—4,7-dimethyl-benzofuran; 30—ethyl 2-methyloctanoate; 31—methyl diethyldithiocarbamate; 32—n-decanoic acid; 33—ethyl 9-decenoate; 34—ethyl decanoate; 35—dodecanal; 36—hexathiane; 37—2-methyl-decanoic acid; 38—dodecanoic acid; 39—ethyl dodecanoate; 40—hexyl octanoate; 41—tetradecanal; 42—octyl ether; 43—2-dodecen-1-ol; 44—ethyl tetradecanoate; 45—hexadecanal; 46—9-hexadecenoic acid; 47—cyclic octaatomic sulfur; 48—ethyl hexadecanoate.

In order to ascertain whether the elevated concentration of added thyme EO had an impact on the profile of meat-derived volatiles, a principal component analysis (PCA) was conducted (Figure 1a). The results demonstrated that the profiles of fresh and stored control samples were distinct from those with EOs. The samples containing EO were divided into two groups: one comprising meat with an addition of 0.005 and 0.01% *v*/*w*, and the other with meat containing 0.02 and 0.03% *v*/*w*. This indicates a pronounced modifying effect of the essential oil on the odour components occurring in the meat itself, and a relatively small difference between the pairs of the aforementioned EO concentrations. Furthermore, the results presented in Figure 1b demonstrate that all detected meat-derived components can be divided into three distinct groups. The first group, corresponding to fresh meat (Figure 1a), is comprised primarily of compounds with low LRI, including aldehydes such as hexanal, octanal, 2-pentylfuran, octenal, and benzaldehyde, as well as some alcohols, such as 1-hexanol. The second group, which corresponds to stored meat with added EO, is represented by esters. The third group, corresponding to stored meat without thyme EO, comprises a more diverse group of compounds, including those containing sulphur.

**Figure 2:**
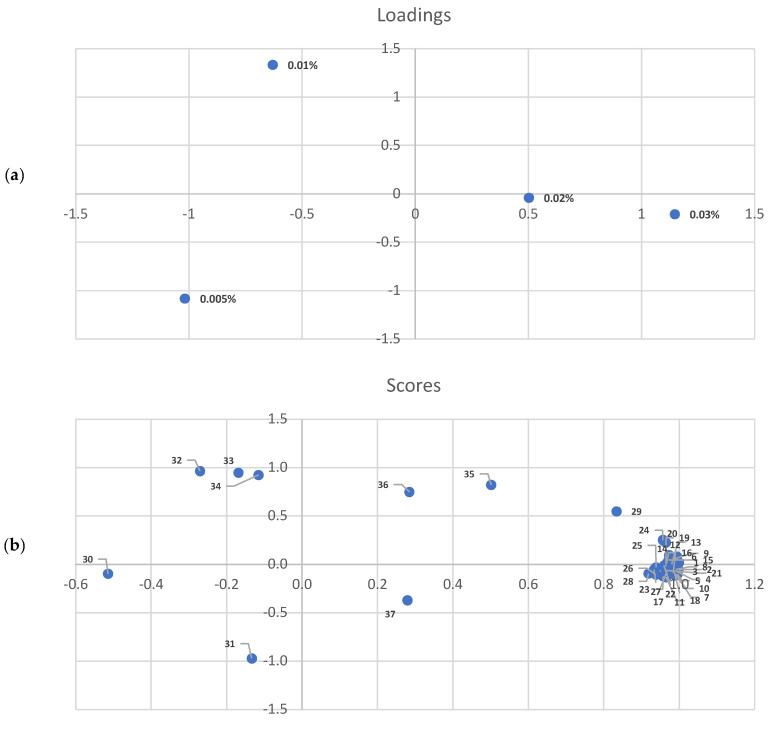
Principal component analysis (PCA) scores and loadings plots of the volatile compounds from thyme essential oil. (**a**) 0.005%, 0.01%, 0.02%, 0.03%—concentration of thyme essential oil in samples; (**b**), 1—α-phellandrene; 2—isothymol methyl ether; 3—β-myrcene; 4—terpinolene; 5—δ-cadinene; 6—α-terpinene; 7—β-pinene; 8—α-pinene; 9—camphene; 10—humulene; 11—trans-β-ocimene; 12—cis-β-ocimene; 13—thujene; 14—caryophyllene oxide; 15—carvacrol; 16—thymol; 17—α-copaene; 18—caryophyllene; 19—γ-terpienene; 20—limonene; 21—isocaryophyllene; 22—cymene; 23—linalool; 24—cymenene; 25—borneol; 26—terpinen-4-ol; 27—terpineol; 28—linalool oxide; 29—aromadendrene; 30—γ-muurolene; 31—germacrene D; 32—carvone; 33—eugenol; 34—caryophyllene alcohol; 35—camphore; 36—α-muurolene; 37—β-farnesene.

Figure 2 presents the results of the principal component analysis of the EO-derived components. In contrast to previous observations, samples with 0.005 and 0.01% EO addition exhibited a high degree of diversification. The differences were more pronounced than those observed at concentrations of 0.02 and 0.03% (Figure 2a). As illustrated in Figure 2b, the distribution of individual components indicates a correlation between the concentration of a given component and the concentration of added EO. Nevertheless, some components, such as carvone, were more prevalent in samples with a lower EO addition (0.01%).

### 2.3. Microbial and Rancidity Indicators and Odour Assassment

The odour of meat is influenced by two distinct factors: the microorganisms present in the meat, which are responsible for the production of, e.g., esters, and the oxidation products of fat. The results obtained for the relevant indicators are presented in Table 4. The meat samples exhibited moderate initial microbiological contamination, which increased by approximately 2 log cycles following the storage period (Table 4). The addition of essential oil had no beneficial effect on the total microbial count, regardless of the concentration used. Thyme essential oil and its constituent thymol are well known for their antimicrobial properties, with numerous studies confirming these effects in vitro [18]. In certain studies, the dosage applied, for instance, 0.125%, was also found to affect evaluated microorganisms, with a reduction in their number and an extension of shelf life [33]. However, in some cases, even high doses were ineffective. For example, Solomakos et al. [34] added 0.6% thyme EO to minced beef meat and found no inhibitory effect on *Escherichia coli* O157:H7 for samples stored at 4 °C. This effect was only observed at a storage temperature of 10 °C [34]. The efficacy of essential oils as preservatives is influenced by, among other things, the characteristics of the products themselves, the number and type of micro-organisms present, and the conditions under which the products are stored. These factors may limit the effectiveness of the measures used [20].

Each of the EO concentrations used improved the sensory evaluation of the odour. In the stored product, as the amount of EO added increased, the sum of the concentrations of the EO-derived components increased from 1670 (0.005%) to 11,139 (0.03%) μg·kg^−1^ of meat. The relationship between odour assessment results and EO concentration was strongly linear (r = 0.92). The positive effect of EO addition on the odour of stored meat products has also been reported in other studies. Karabagias et al. [35] found that the addition of thyme EO had a beneficial effect on the odour of minced lamb and prolonged its shelf life. Chouliara et al. [36] came to similar conclusions when evaluating the effect of oregano essential oil on the shelf-life of fresh chicken breast meat. In addition, Karam et al. [37], who marinated raw chicken breast fillets with thymol and carvacrol, confirmed that the odour acceptability of a product can be extended for the next few days.

**Figure 3:**
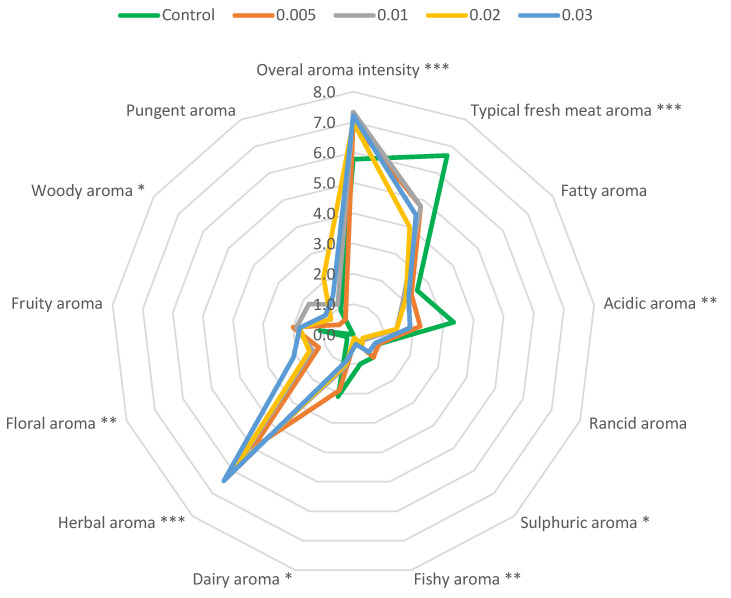
The spider web diagrams visualise the aroma qualities of stored, vacuum-packed minced turkey meat without and with different concentrations of thyme EO added. *, **, ***—the significance at 0.05, 0.01, and 0.005 by least significant difference, respectively.

Quantitative descriptive analysis (QDA) showed a statistically significant effect of the addition of various concentrations of thyme EO on 9 of the 13 quantities analysed (Figure 3). Five of these were reduced—typical fresh meat aroma, acidic, sulphuric, fishy, and dairy. They were related to turkey meat aroma and subsequent microbiological, enzymatic, and physical changes in the meat during storage. The concentration of EO added had no statistical effect on the values of these parameters. The remaining four qualities, namely, overall aroma intensity, and herbal, floral, and woody aroma, increased as the amount of EO added increased. At the highest EO concentration used (0.03% *v*/*w*), the overall odour intensity was so high that notes similar to an organic solvent appeared. Pleasant citrus notes characterised the top-rated turkey samples (Table 4) containing 0.02% thyme EO. Numerous studies on the addition of herbs and their EOs to meat have shown that their influence on the sensory characteristics, especially the aroma of the product, is significant. Not only do they limit the development of microorganisms and the processes involved in the formation of unpleasant odours, but their high volatile content can also mask the effect of unfavourable sensory changes [3,4,5]. If their concentration is too high, they can cause unpleasant solvent notes as well as various unpleasant aftertastes and bitterness. It is therefore important to determine the dose of EO in order to obtain the most positive effect without negatively affecting the sensory properties of the final product [38].

The addition of essential oil had a positive effect on the acid and peroxide values and on the TBARS indicator. For the latter indicator (r = −0.93) and for AV (r = −0.93), there was a clear relationship between the amount of EO and the value of the indicator evaluated in the stored meat. The relationship was weaker for the peroxide value (r = −0.73). Meat mincing promotes fat rancidity both by introducing oxygen into the product, creating a larger area of fat deposition, and by destroying tissue, facilitating contact between enzymes and their substrates, and dispersing microorganisms throughout the mass. It also facilitates the contact of unsaturated fatty acids with non-haem iron, a pro-oxidant [17]. Wong et al. [39] reported that the content of polyunsaturated fatty acids in retail minced turkey meat, expressed as a weight percentage of total fatty acid methyl esters, ranged from 24.6 to 32.5%, while the fat content ranged from 7.2 to 10.8%. In turkey meat, a relatively low number of tocopherols is also an important factor favouring the oxidation process [40]. According to Jayasena and Jo [17], antioxidant substances in turkey meat include ascorbic acid (9 mg·kg^−1^ of dark muscle) and glutathione (90 mg·kg^−1^ of thigh). In this study, the AV, PV, and TBARS values were indicators of the changes occurring in the fats. The acid value indicates the degree of fat hydrolysis and the concentration of free fatty acids. The peroxide value, in turn, determines the concentration of peroxides, which are unstable and then converted to carbonyl compounds, as assessed by the TBARS indicator. This indicator is used to determine malondialdehyde, which is a secondary product of lipid oxidation. However, other substances, including other low-molecular-weight aldehydes such as 2,4-alkadienals and peptides, may also react with TBA [41,42]. Boskovic et al. [12] used this indicator to evaluate the antioxidant effect of thyme and oregano essential oils added to minced pork. The authors found that the addition of EOs increased the stability of the studied meat in terms of lipid oxidation and that the antioxidant effect depended on the dose applied [12].

Some of the aldehydes formed in these reactions can also be used as indicators of fat oxidation. Hexanal and nonanal, among others, are sometimes used for this purpose [43]. Sums of selected volatile compounds have also been proposed as markers of rancidity for olive oil [44]. However, in the present study, no correlation was found between PV and TBARS values and the content of sum of meat-derived volatile compounds, sum of carbonyl compounds, and nonanal and hexanal.

The main constituent of the thyme essential oil evaluated in this study was thymol. Although carvacrol was also found, its contribution was much lower (Table 1). As reported by Tohidi et al. [45], thymol is a major constituent of the essential oil of most plants belonging to the Thymus species. The results of Yanishlieva et al. [46] showed that both compounds had antioxidant properties. Of the two substances, thymol was more effective and active as an antioxidant due to the greater steric hindrance of the phenolic group. In addition, the authors noted that unlike carvacrol radicals, which were involved in one reaction of chain propagation during lard and sunflower oil systems oxidation, thymol radicals were not [44]. The remaining components of thyme EO may also influence its antioxidant and pro-oxidant properties. In general, our studies indicate a beneficial effect of the thyme EO used on the stability of turkey fat, even at concentrations as low as 0.005% *v*/*w*.

## 3. Materials and Methods

### 3.1. Raw Material and EO Addition

The raw material was turkey knuckle meat. The meat was purchased at the local market 24 h after slaughter. The meat was minced (mesh diameter 4 mm) and divided into five batches. The control samples contained no additives. The others were mixed with thyme EO at concentrations of 0.005%, 0.01%, 0.02%, and 0.03% *v*/*w*. The 0.4 kg portions of the products (3 of each type) were vacuum-packed (Vac-Star 1000, Sugiez, Switzerland) and stored at 1–2 °C in the dark. The essential oil doses were chosen on the basis of preliminary analyses. The dose of 0.02% *v*/*w* was the maximum dose accepted by all sensory panelists, while the dose of 0.03% *v*/*w* was only accepted by some panelists. Analyses were performed on fresh product and after 8 days of storage. The fat content was determined by the Soxhlet method and was 6.4%. The water and ash contents were 75.13% and 0.99%, respectively. The protein content, determined by the Kjeldahl method, was 17.7%.

### 3.2. Analysis of Essential Oils and Volatile Compounds

Commercially available 100% natural thyme essential oil and turkey meat, with or without added EO, were analysed by headspace solid-phase microextraction and gas chromatography coupled to time-of-flight mass spectrometry (SPME-GC-TOFMS). For the determination of volatiles in EO, 2 mL of saturated saline, 0.1 mL of internal standard solution (5 mg·L^−1^ 4-methyl-2-pentanol, 0.05 mg·L^−1^ ethyl nonanoate and 0.5 mg·L^−1^ anethol, Sigma-Aldrich), and a 10 µL sample of EO were added to a 10 mL vial. For turkey samples, the sample was prepared as above, but instead of the EO, a 0.5 g sample of turkey meat was placed in a 10 mL vial. The SPME device (Supelco Inc., Bellefonte, PA, USA) coated with PDMS fibre (100 μm) was first conditioned (250 °C for 1 h) and then placed in the headspace under stirring (300 rpm) at 60 °C for 30 min. The SPME device was then introduced into the injector port of the Agilent Technologies 7890B chromatograph system equipped with LECO Pegasus HT, High Throughput TOFMS, with GERSTEL MultiPurpose Sampler (MPS) and held in the inlet for 3 min. The chromatographic separation was performed in the splitless mode on the Rxi^®^-1ms capillary column (Crossbond 100% dimethyl polysiloxane, 30 m × 0.25 mm × 0.25 µm; Restek, Bellefonte, PA, USA). Detection was performed as previously described [30].

Mass spectra were recorded in SEM mode. Compound identification was performed using mass spectral libraries (NIST) and linear retention indices derived from the C6 to C20 n-alkane series. Qualitative and quantitative identification of volatiles (components in Table 1, Table 2 and Table 3; Sigma-Aldrich, Darmstadt, Germany) was based on comparison of retention times and peak areas of sample and standard chromatograms. Other detected components were determined semi-quantitatively (μg·kg^−1^) by measuring the relative peak area of each identified compound, in relation to that of the chemically similar standard.

Changes in the content of the analysed volatile compounds derived from thyme EO are shown in Table 3 in the form of corresponding colours. The components whose content increased during storage are marked in various shades of green, while those whose content decreased during storage are marked in various shades of red.

### 3.3. Microbial and Fatty Rancidity Indicators Analysis

The total viable count (TVC) was determined on plate count agar (PCA) incubated at 30 °C for 72 h according to the Polish standard (PN-EN ISO 4833-1:2013-12).

Acid (AV) and peroxide (PV) values were determined according to standard methods, namely, Cd 3d-63 and Cd 8b-90, respectively [47]. The content of thiobarbituric acid reactive substances (TBARS) was determined by an extraction method, measuring the absorbance of the red coloured complexes at 532 nm [48]. The results, expressed as mg malondialdehyde (MDA) per kg fat, were calculated from the standard curve of the 1,1,3,3-tetraethoxypropane standard (Sigma-Aldrich, Darmstadt, Germany).

### 3.4. Odour Analysis

The odour of raw meat was scored according to a pre-established table. A score of 5 points was given to the highest quality, 4 points to good quality, and 2 points to the poorest (and unacceptable) quality samples. The acceptability threshold was set at 3 points. The evaluation was carried out by a sensory panel of 7 members, trained and proven in sensory sensitivity.

The turkey samples also underwent aroma assessment using quantitative descriptive analysis (QDA). The evaluation was carried out by a panel of 10 trained individuals, comprising 5 males and 5 females, aged between 30 and 50 years, employed by the Department of Fermentation Technology and Microbiology and Department of Biotechnology and General Technology of Food. Thirteen aroma qualities were rated on a ten-point scale, including overall aroma intensity, typical fresh meat aroma, fatty, acidic, sulphuric, rancid, fishy, dairy, herbal, floral, fruity, woody, and pungent aroma.

### 3.5. Statistical Analysis

Analyses were carried out in triplicate. The charts present the results as arithmetic means with standard deviation (SD; Table 1) or standard error of the mean (SEM) (Table 2, Table 3 and Table 4). The significance of differences between means was determined using one-way analysis of variance and Tukey’s test (Table 2, Table 3 and Table 4). PCA analysis was performed using SPSS version 23 software (Chicago, IL, USA).

## 4. Conclusions

Thyme essential oil at any of the concentrations used did not affect the overall microbiological contamination, whereas each of the concentrations used, even that of 0.005% *v*/*w*, reduced the increase in at least one of the fat rancidity indicators measured. At all concentrations, the evaluated additive had a significant modifying effect on the profile of meat-derived volatile compounds. The PCA analysis showed a clear distinction between the profiles of meat-derived volatiles of the samples with EO addition and the fresh and stored control samples. At the same time, the results suggest similarities in the profiles of meat with 0.005 and 0.01% EO addition and those with 0.02 and 0.03% EO addition. However, further research is needed to determine the mechanisms of transformation of individual components of both meat and essential oils during storage and their direct impact on the aroma of meat after storage.

## Figures and Tables

**Table 1:** Percentage of the main volatile components in the thyme essential oil used in the experiments.

Compound	Ion [*m*/*z*]	LRI		Content [%]	SD ^1^
		Exp ^a^	Lit ^b^		
α-Pinene	93	935	933	1.4	0.3
Camphene	93	951	952	1.6	0.3
β-Pinene	93	980	978	0.4	0.2
β-Myrcene	41	984	985	2.9	0.6
α-Phellandrene	93	994	994	1.4	0.2
α-Terpinene	121	1006	1005	4.0	0.0
p-Cymene	119	1010	1013	5.3	0.8
D-Limonene	68	1029	1026	1.3	0.3
(Z)-β-Ocimene	93	1034	1032	1.3	0.3
γ-Terpinene	93	1049	1050	6.2	0.9
p-Cymenene	117	1067	1069	0.5	0.0
Terpinolene	93	1081	1081	1.2	0.2
Linalool	71	1084	1086	4.2	0.5
Camphor	95	1114	1115	3.1	0.0
Borneol	95	1145	1142	10.0	0.1
Terpinen-4-ol	71	1158	1158	3.3	0.7
α-Terpineol	59	1169	1172	5.9	0.7
Thymol	135	1294	1287	23.1	2.0
Carvacrol	135	1296	1295	4.0	0.5
α-Copaene	161	1376	1377	1.3	0.4
Isocaryophyllene	41	1403	1407	1.4	0.1
Caryophyllene	93	1419	1422	6.3	0.3
Aromandendrene	41	1438	1439	1.2	0.1
Humulene	93	1457	1454	3.0	0.2
γ-Muurolene	161	1471	1466	0.6	0.1
δ-Cadinene	161	1514	1514	0.9	0.1
Other				4.3	0.2

^a^ LRI exp—linear retention indices measured on an Rxi^®^-1ms column; ^b^ LRI lit—reference LRI values from the National Institute of Standards and Technology (NIST) 2023 RI Database for column and separation conditions most similar to those used in experiments; ^1^ SD—standard deviation.

**Table 2:** Volatile compounds derived from thyme essential oil after storage of meat samples and their changes compared to values obtained immediately after addition to the meat.

Compound (μg·kg^−1^)			Ion [*m*/*z*]	LRI		Stored Meat				SEM ^1^	Sig. ^2^
				Exp ^a^	Lit ^b^	Concentration of Added Thyme Essential Oil (% *v*/*w*)					
						0.005	0.01	0.02	0.03		
α-Thujene ^x^			93	928	926	1.1 a	3.5 a	12.7 b	13.7 b	1.4	***
α-Pinene			93	935	933	2.8 a	5.2 a	18.5 b	21.8 b	2.3	***
Camphene			93	951	952	4.4 a	8.7 a	19.0 b	21.8 b	2.1	***
β-Pinene			93	980	978	1.5 a	1.9 a	5.5 b	6.7 b	0.6	***
β-Myrcene			41	984	985	5.1 a	8.9 a	29.1 b	42.8 c	4.0	***
α-Phellandrene			93	994	994	3.1 a	7.1 a	16.7 b	23.5 c	2.2	***
α-Terpinene			121	1006	1005	3.4 a	10.1 a	23.5 b	28.5 b	2.8	***
p-Cymene			119	1010	1013	145 a	276 a	913 b	895 b	96.7	***
D-Limonene			68	1029	1026	8.5 a	63.2 b	95.2 c	149.4 d	13.7	***
(Z)-β-Ocimene			93	1034	1032	2.2 a	2.7 a	5.6 b	8.6 c	0.7	***
(E)-β-Ocimene ^x^			93	1038	1043	2.2 a	3.7 a	11.2 b	18.7 c	1.7	***
γ-Terpinene			93	1049	1050	19.4 a	49.9 a	203.3 b	203.5 b	22.8	***
cis-Linalool oxide			59	1058	1059	1.4 a	2.0 ab	4.1 b	10.1 c	0.9	***
p-Cymenene			117	1067	1069	3.2 a	6.5 ab	9.8 bc	10.5 c	1.1	**
Terpinolene			93	1081	1081	4.6 a	6.4 a	16.1 b	23.1 c	2.1	***
Linalool			71	1084	1086	42.2 a	56.8 a	183.0 b	378.2 c	33.1	***
Camphor			95	1114	1115	141.8	240.5	194.4	232.0	25.5	ns
Borneol			95	1145	1142	59.9 a	115.2 b	206.2 c	453.6 d	38.5	***
Terpinen-4-ol			71	1158	1158	22.9 a	34.6 a	92.3 b	216.4 c	18.6	***
α-Terpineol			59	1169	1172	22.8 a	42.0 a	81.0 b	188.6 c	16.1	***
Carvone			82	1243	1242	57.3 a	579.9 b	193.9 a	118.2 a	51.8	***
Isothymol methyl ether ^x^			149	1244	1244	6.9 a	16.0 a	55.6 b	80.6 b	7.8	***
Thymol			135	1294	1287	918 a	2158 a	4025 b	7544 c	656.8	***
Carvacrol			135	1297	1295	129.7	142.9	159.9	190.2	18.7	ns
Eugenol			164	1351	1345	1.0 a	6.4 ab	3.9 b	1.6 a	0.6	***
α-Copaene			161	1376	1377	1.0 a	1.3 a	4.4 b	4.6 b	0.5	***
Isocaryophyllene ^x^			41	1403	1407	1.4 a	1.7 a	4.6 b	4.6 b	0.5	***
Caryophyllene			93	1419	1422	38.2 a	41.6 a	184.5 b	194.4 b	20.8	***
Aromadendrene ^x^			41	1437	1439	0.5 a	1.4 ab	1.5 b	1.7 b	0.2	*
Humulene			93	1448	1454	6.4 a	7.6 a	25.4 b	31.0 b	3.0	***
β-Farnesene			41	1451	1451	0.4	0.2	0.7	0.3	0.1	ns
Germacrene D ^x^			161	1475	1480	1.3	0.4	0.7	0.9	0.1	ns
γ-Muurolene ^x^			161	1473	1466	0.6	0.6	0.4	0.5	0.1	ns
α-Muurolene ^x^			105	1497	1493	0.2	0.3	0.2	0.2	0.0	ns
δ-Cadinene ^x^			161	1514	1514	1.4 a	1.7 a	4.7 b	6.4 b	0.6	***
Caryophyllene alcohol ^x^			109	1560	1557	0.8 a	1.9 b	1.5 ab	1.0 a	0.2	**
Caryophyllene oxide ^x^			43	1570	1575	6.4 a	8.4 b	12.7 c	13.1 d	1.3	***
>3	2 to 3	1 to 2	0 to 1	0 to −0.25		−0.25 to −0.5		−0.5 to −0.75		<−0.75	

^a^ LRI exp—linear retention indices measured on an Rxi^®^-1ms column; ^b^ LRI lit—reference LRI values from the National Institute of Standards and Technology (NIST) 2023 RI Database for column and separation conditions most similar to those used in experiments. The numerical values in the table refer to the concentration of volatile components after storage time of meat samples. Changes in the content of individual components, relative to the level in meat at the time of adding EO, are marked with colours, ranging from over threefold increase (dark green) to over 0.75-fold reduction (dark red); ^1^ SEM—standard error of means; ^2^ Sig.—significance; *, **, ***—display the significance at 0.05, 0.01, and 0.005 by least significant difference, respectively; ns: not significant. Values with different letters (a–d) in the same row are significantly different according to the Tukey’s test (*p* < 0.05); ^x^—determined semi-quantitatively by measuring the relative peak area of each identified compound, according to the NIST database, in relation to that of the chemically similar standard.

**Table 3:** Volatile compounds derived from vacuum-packed, chill-stored turkey meat.

Volatile Compounds (μg·kg^−1^)	Ion (*m*/*z*)	LRI		Fresh Meat	Stored Meat					SEM ^1^	Sig. ^2^
		Exp ^a^	Lit ^b^		Concentration of Added Thyme Essential Oil (% *v*/*w*)						
					0	0.005	0.01	0.02	0.03		
**Carbonyl compounds**											
Acetaldehyde	29	367	363	3.9 a	5.9 ab	15.3 ab	17.1 ab	24.0 b	62.2 c	4.9	***
Hexanal	44	800	801	139 c	42 b	28 b	3 a	2 a	2 a	10.8	***
Heptanal	70	904	902	3.5 b	0.3 a	1.2 ab	0.0 a	0.0 a	0.0 a	0.4	***
Benzaldehyde	77	925	924	5.1	2.4	2.5	1.5	0.8	2.1	0.5	ns
2-Heptenal ^x^	41	936	941	4.7 b	0.8 a	0.5 a	0.0 a	0.0 a	0.0 a	0.5	***
1-Octen-3-one	55	953	958	11.9 b	2.4 a	0.7 a	0.3 a	0.0 a	0.0 a	1.1	***
Octanal	43	982	984	6.8 b	2.0 a	1.2 a	1.0 a	1.3 a	2.3 a	0.6	***
Benzeneacetaldehyde	91	1004	1005	0.0 a	18.3 c	9.3 b	5.6 ab	0.0 a	2.5 ab	1.7	***
2-Octenal ^x^	41	1035	1042	7.4 c	1.2 b	0.0 a	0.0 a	0.0 a	0.0 a	0.7	***
Nonanal	57	1083	1083	17.7 c	7.7 b	3.1 ab	0.3 a	0.0 a	0.0 a	1.8	***
Decanal	43	1188	1191	0.9	2.6	1.6	2.5	9.2	8.1	0.9	ns
Dodecanal	57	1388	1386	1.5 a	5.8 b	3.3 ab	3.2 ab	2.5 a	2.4 a	0.4	***
Tetradecanal ^x^	57	1590	1588	2.7	5.2	4.3	4.3	2.0	3.9	7.0	ns
Hexadecanal ^x^	82	1830	1822	32	49	74	62	16	45		ns
**Alcohols**											
1-Pentanol	42	768	773	13.1 c	4.9 b	4.0 b	0.0 a	0.0 a	0.0 a	1.1	***
1-Hexanol	56	852	853	6.0 b	2.4 a	0.9 a	0.0 a	0.0 a	0.0 a	0.6	***
1-Octen-3-ol	57	963	959	0.8	1.0	0.9	0.4	1.1	1.4	0.1	ns
Benzyl alcohol	79	1036	1033	0.7 a	23.3 c	3.0 ab	11.3 b	8.4 ab	7.8 ab	1.9	***
2-Octen-1-ol ^x^	57	1058	1060	5.1 b	1.5 ab	0.3 a	0.0 a	0.0 a	0.0 a	0.6	**
2-Dodecen-1-ol ^x^	57	1675	1680	1.9	2.4	3.8	2.7	1.1	2.0	0.3	ns
**Acids**											
Hexanoic acid	60	975	973	0.0 a	6.3 c	4.6 bc	4.0 bc	2.4 ab	0.0 a	0.6	***
Octanoic acid	60	1162	1162	2.2 a	4.4 ab	6.5 a	6.7 ab	6.3 a	11.1 b	0.7	***
n-Decanoic acid	60	1354	1359	0.0 a	3.4 ab	9.1 abc	12.6 abc	17.8 bc	19.3 c	1.9	**
2-Methyldecanoic acid ^x^	74	1500	na	0.0 a	0.5 ab	2.6 d	1.4 bc	2.6 cd	2.2 cd	0.3	***
Dodecanoic acid	73	1547	1548	6.9 a	9.3 a	28.8 ab	30.4 ab	45.1 ab	59.3 b	5.7	*
9-Hexadecenoic acid	55	1910	1916	0.0 a	0.0 a	5.1 b	3.0 b	4.3 bc	3.7 b	0.5	***
**Esters**											
Ethyl acetate	43	614	612	0.0 a	0.5 ab	2.0 c	1.4 bc	1.8 c	2.5 c	0.2	***
Ethyl octanoate	88	1182	1179	4.6 b	1.2 a	1.2 a	1.2 a	3.8 b	3.3 ab	0.4	*
Ethyl 2-methyloctanoate	102	1231	1225	43.0 a	129.9 c	29.7 a	41.7 a	78.3 b	46.8 a	8.6	***
Methyl diethyldithiocarbamate ^x^	163	1349	1357	1.9 a	4.5 ab	6.1 b	2.6 a	5.0 ab	4.4 ab	0.4	**
Ethyl 9-decenoate	88	1370	1370	0.0 a	0.0 a	1.0 c	0.4 ab	0.8 bc	0.8 bc	0.1	***
Ethyl decanoate	88	1384	1377	6.6 a	11.2 ab	22.0 bc	14.7 abc	27.2 c	22.3 bc	2.0	***
Ethyl dodecanoate	88	1576	1577	17.5 a	34.6 a	115.3 bc	56.2 ab	124.6 c	111.4 bc	11.1	***
Hexyl octanoate	43	1582	1584	7.3 ab	12.8 ab	17.0 b	6.1 a	6.2 a	10.4 ab	1.2	*
Ethyl tetradecanoate	88	1777	1780	1.5 a	2.4 a	9.4 b	4.3 a	8.5 b	8.5 b	0.8	***
Ethyl hexadecanoate	88	1975	1975	0.8 a	1.4 ab	4.1 b	1.6 ab	3.1 ab	3.4 ab	0.4	*
**Others**											
3-Propoxy-1-propene ^x^	58	691	na	7.8 c	2.6 b	3.1 b	0.6 a	0.0 a	0.0 a	0.7	***
Dimethyl disulphide	94	729	731	0.0	2.6	1.2	1.3	1.3	1.9	0.2	ns
Butyrolactone	42	870	870	0.0 a	0.0 a	14.0 b	8.8 ab	7.4 ab	13.5 b	1.7	**
Dimethyl sulfone ^x^	79	892	914	0.0 a	0.0 a	0.0 a	0.0 a	29.0 b	32.2 b	4.2	***
Dimethyl trisulphide	126	949	949	1.6	1.4	0.6	1.8	1.0	1.2	0.1	ns
2-Pentylfuran	81	980	982	27.5 b	4.8 a	2.6 a	0.8 a	0.3 a	0.0 a	2.4	***
Dimethyl tetrasulphide	79	1186	1192	0.0	0.0	0.0	0.4	0.6	1.4	0.1	ns
4,7-Dimethylbenzofuran ^x^	146	1220	1220	7.9 a	44.6 b	7.8 a	14.2 a	12.0 a	18.5 ab	3.2	*
Hexathiane^x^	192	1440	1499	5.8 ab	17.0 b	4.2 a	12.0 ab	5.9 a	4.2 a	1.4	**
Octyl ether^x^	57	1657	1660	4.6	4.1	5.3	5.2	4.6	2.8	0.3	ns
Cyclic octaatomic sulphur^x^	64	1959	1998	6.6	10.4	4.4	7.8	12.3	3.6	1.0	ns

^a^ LRI exp—linear retention indices measured on an Rxi^®^-1ms column; ^b^ LRI lit—reference LRI values from the National Institute of Standards and Technology (NIST) 2023 RI Database for column and separation conditions most similar to those used in experiments; na—LRI not available in published databases; ^1^ SEM—standard error of means; ^2^ Sig.—significance; *, **, ***—the significance at 0.05, 0.01, and 0.005 by least significant difference, respectively; ns: not significant. Values with different letters (a–d) in the same row are significantly different according to the Tukey’s test (*p* < 0.05); ^x^—determined semi-quantitatively by measuring the relative peak area of each identified compound, according to the NIST database, in relation to that of the chemically similar standard.

**Table 4:** Changes in the number of microorganisms (log cfu·g^−1^), odour of raw meat (scores), and lipid quality indicators of raw vacuum-packed minced turkey meat with or without the addition of essential oil at different concentrations (% *v*/*w*), stored at 1–2 °C.

Indicator	Fresh Meat	Stored Meat					SEM ^1^	Sig. ^2^
		Concentration of Added Thyme Essential Oil (% *v*/*w*)						
		0	0.005	0.01	0.02	0.03		
Total viable count	5.33 a	7.68 b	7.70 b	7.88 b	7.60 b	7.92 b	0.22	ns
AV [mg·g^−1^]	3.22 ad	3.95 c	3.79 c	3.59 bcd	3.12 a	3.20 ab	0.08	***
PV [meq·kg^−1^]	2.74 a	3.80 b	2.88 a	2.92 a	2.75 a	2.71 a	0.09	***
TBARS [mg MDA·kg^−1^]	1.55 ac	2.00 b	1.87 ab	1.69 ab	1.09 d	1.17 cd	0.09	***
Odour of raw meat [pts]	5.0 a	2.3 b	2.8 bc	3.1 c	3.0 c	4.0 d	0.2	***

Values in the row regarding the same parameter, marked with different letters, differ statistically significantly (*p* < 0.05); ***—the significance at 0.005 by least significant difference; ns—not significant; ^1^ SEM—standard error of means; ^2^ Sig.—significance.

## Data Availability

Data are contained within the article.
